# Impact of coronary bifurcation angle on computed tomography derived fractional flow reserve in coronary vessels with no apparent coronary artery disease

**DOI:** 10.1007/s00330-022-09125-3

**Published:** 2022-09-17

**Authors:** Toshimitsu Tsugu, Kaoru Tanaka, Yuji Nagatomo, Dries Belsack, Hannes Devos, Nico Buls, Bernard Cosyns, Jean-François Argacha, Michel De Maeseneer, Johan De Mey

**Affiliations:** 1 Department of Radiology, Universitair Ziekenhuis Brussel, Laarbeeklaan 101, 1090 Jette, Brussels, Belgium; 2 Department of Cardiology, National Defense Medical College Hospital, Tokorozawa, Japan; 3 Cardiology, Centrum voor Hart-en Vaatziekten, Universitair Ziekenhuis Brussel, Brussels, Belgium

**Keywords:** FFRCT, Angulation, Coronary computed tomography, Fractional flow reserve, Coronary artery disease

## Abstract

**Objectives:**

Computed tomography (CT) derived fractional flow reserve (FFR_CT_) decreases from the proximal to the distal part due to a variety of factors. The energy loss due to the bifurcation angle may potentially contribute to a progressive decline in FFR_CT_. However, the association of the bifurcation angle with FFR_CT_ is still not entirely understood. This study aimed to investigate the impact of various bifurcation angles on FFR_CT_ decline below the clinically crucial relevance of 0.80 in vessels with no apparent coronary artery disease (CAD).

**Methods:**

A total of 83 patients who underwent both CT angiography including FFR_CT_ and invasive coronary angiography, exhibiting no apparent CAD were evaluated. ΔFFR_CT_ was defined as the change in FFR_CT_ from the proximal to the distal in the left anterior descending artery (LAD) and left circumflex artery (LCX). The bifurcation angle was calculated from three-dimensional volume rendered images. Vessel morphology and plaque characteristics were also assessed.

**Results:**

ΔFFR_CT_ significantly correlated with the bifurcation angle (LAD angle, *r* = 0.35, *p* = 0.001; LCX angle, *r* = 0.26, *p* = 0.02) and vessel length (LAD angle, *r* = 0.30, *p* = 0.005; LCX angle, *r* = 0.49, *p* < 0.0001). In LAD, vessel length was the strongest predictor for distal FFR_CT_ of ≤ 0.80 (*β*-coefficient = 0.55, *p* = 0.0003), immediately followed by the bifurcation angle (β-coefficient = 0.24, p = 0.02). The bifurcation angle was a good predictor for a distal FFR_CT_ ≤ 0.80 (LAD angle, cut-off 31.0°, AUC 0.70, sensitivity 74%, specificity 68%; LCX angle, cut-off 52.6°, AUC 0.86, sensitivity 88%, specificity 85%).

**Conclusions:**

In vessels with no apparent CAD, vessel length was the most influential factor on FFR_CT_, directly followed by the bifurcation angle.

**Key Points:**

*• Both LAD and LCX bifurcation angles are factors influencing FFR*_*CT*_*.*

*• Bifurcation angle is one of the predictors of a distal FFR*_*CT*_ *of ≤ 0.80 and an optimal cut-off value of 31.0° for the LAD and 52.6° for the LCX.*

*• Bifurcation angle should be taken into consideration when interpreting numerical values of FFR*_*CT*_ *.*

**Supplementary Information:**

The online version contains supplementary material available at 10.1007/s00330-022-09125-3.

## Introduction

FFR_CT_ is a reliable method for detecting functional ischemia, but it can be difficult to interpret because it is affected by a variety of factors such as vessel length [1], plaque characteristics [2, 3], left ventricular mass [4, 5], ramus artery [6], or collateral circulation [7]. The left coronary artery bifurcation angle has been shown to affect wall shear stress and cause modifications to the bloodstream [8, 9]. Significant modifications of laminar flow can contribute to FFR values when taking into account well-established factors such as lumen narrowing and lesion length [10]. We previously reported that diverse FFR_CT_ changes at distal segments occur due to the differences in the left coronary bifurcation angle even with the same vessel length and plaque characteristics [11]. The left coronary artery bifurcation angle contributes to hemodynamic changes in the left coronary arteries and may affect the FFR_CT_. However, the effect of the left coronary artery bifurcation angle on FFR (FFR_CT_ and invasive FFR) has not been clarified. The present study aimed to identify the effect of atypical left coronary artery bifurcation angle on an FFR_CT_ decline below the clinically relevant value of 0.80 in vessels with no apparent CAD.

## Methods

### Patient population

**Fig. 1:**
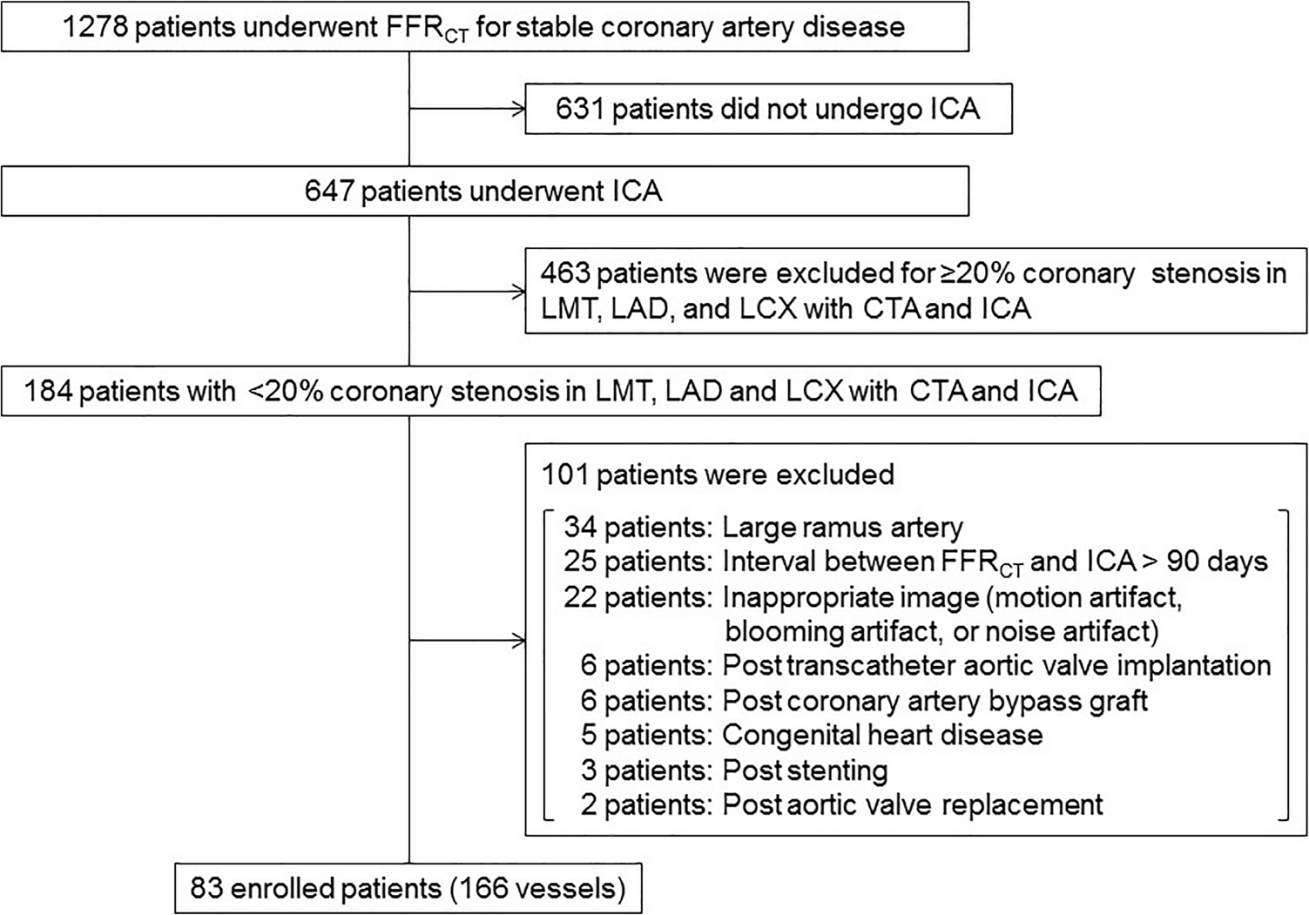
Patient selection flow diagram showing patient flow in the study. CTA, CT angiography; FFR_CT_, CT derived fractional flow reserve; ICA, invasive coronary angiography; LAD, left anterior descending artery; LCX, left circumflex artery; LMT, left main trunk

A total of 1278 outpatients with suspected coronary artery disease (CAD) and who had a CT angiography (CTA) with FFR_CT_ analysis examined at the Universitair Ziekenhuis Brussel between January 2017 and March 2021 were evaluated. A retrospective case-control cohort analysis was conducted with approval from the research ethics board at the Universitair Ziekenhuis Brussel under protocol number B.U.N. 14320. The main inclusion criteria were normal vessels or vessels with no apparent CAD on invasive coronary angiography (ICA) and CTA in the left main trunk, left anterior descending artery (LAD) and left circumflex artery (LCX). FFR_CT_ of other vessels, such as the right coronary artery or side branches (diagonal and obtuse side branches) were not considered. No apparent CAD was defined as vessels with < 20% coronary stenosis or luminal irregularities [12]. The severity of coronary stenosis was assessed by experienced interventional cardiologists (B.C., JF.A.) using ICA and by experts in cardiac radiology imaging (T.T., K.T.) using CTA. A total of 631 patients who had not undergone ICA and 463 patients who had coronary stenosis with ICA and CTA were excluded from the study. The following categories of patients were also excluded from the study: the large ramus artery that was large enough to be measured by FFR_CT_ (34 patients), the interval between FFR_CT_ and ICA > 90 days (25 patients), inappropriate image quality (motion artifact, blooming artifact, or noise artifact) (22 patients), post-trans-catheter aortic valve implantation (6 patients), post coronary artery bypass graft (6 patients), congenital heart disease (5 patients), post coronary artery stenting (3 patients), post aortic valve replacement (2 patients). Myocardial bridging leads to compression of vessels during the systolic phase, which is prolonged to the diastolic phase, resulting in hemodynamic changes and affecting FFR [13, 14]. Myocardial bridging was absent in our study. In total, 166 vessels including 83 LAD and 83 LCX from 83 patients were enrolled. The patient selection flowchart is presented in Figure 1.

### Coronary computed tomography angiography acquisition

All coronary CT angiography (CTA) scans were acquired with the GE Revolution scanner (GE Healthcare) that had a spatial resolution of 230 μm, a rotational speed of 0.28 s, and a Z-axis coverage of 16 cm enabling to image the heart in one heartbeat. Beta-blockers were administered when necessary for targeting a heart rate of < 60 beats/min. Sublingual nitrates were administered before scanning in all patients. The images were reconstructed at 75 ± 10% of the R-R interval. Coronary arteries were classified based on the American Heart Association classification [15]. Only the major left coronary vessels were considered for analysis and each segment was classified into three categories (proximal, #6 and #11; middle, #7 and #13; distal, #8 and #15).

### FFR_CT_

FFR_CT_ was analyzed by HeartFlow Inc. Computational fluid dynamics and blood flow simulations were performed to calculate FFR_CT_ available at any arbitrary point in the coronary artery. Coronary arteries were classified based on the American Heart Association classification [15] and each segment was divided into three equal segments, proximal, middle, and distal. FFR_CT_ was measured at each point (Supplementary Figure 1A). The magnitude of change in FFR_CT_ (ΔFFR_CT_) was measured from the proximal to the distal at each left coronary artery. A positive FFR_CT_ was defined as a value ≤ 0.80 in accordance with previously published invasive and non-invasive literature [2, 4, 16, 17].

### Left coronary artery bifurcation angle

As in previous studies [18, 19], each bifurcation angle was measured as the crossing angle between the center lines of the left main trunk and each coronary artery (LAD or LCX) on the three-dimensional volume-rendered image of the coronary artery tree. For the left coronary artery bifurcation angle both LAD angle and LCX were determined by assessing the crossing line with the left main trunk (Supplementary Figure 2: center panel).

### Vessel morphology and plaque characteristics

Vessel length, lumen volume, and composition of each vessel were measured using GE AW server 3.2 software (GE Healthcare). Aligning the vessel to the region of interest on FFR_CT_ and vessel length and lumen volume were obtained semi-automatically with Color Code Plaque (GE Healthcare) (Supplementary Figure 1B). Vessel constituents were characterized based on Hounsfield units (HU) into low-attenuation plaque (< 30 HU), intermediate-attenuation plaque (30–150 HU), and calcified plaque (> 150 HU) [20, 21].

### Statistical analysis

Continuous variables were expressed as mean ± standard deviation (SD). The 95% confidence interval (CI) was calculated as ± 1.96 SDs from the mean. Two-group comparisons were performed with unpaired Student’s *t* tests for means if the data were normally distributed or Mann–Whitney *U*-test if the data were not normally distributed. Correlation between continuous variables was performed using the Spearman correlation test. Multivariable linear regression analysis was performed to examine the independent correlations between FFR_CT_ and baseline parameters. A hierarchical cluster analysis (Ward’s method) was performed to classify parameters related to bifurcation angles. Receiver operating characteristics curves were generated to determine the cut-off value that the highest diagnostic performance of an FFR_CT_ ≤ 0.80. All statistical analyses were performed using JMP 11.0 statistical software (SAS Institute).

## Results

### Demographic data

Table 1 summarizes the demographic and CT image acquisition condition data of the study population. The time phase in the cardiac cycle was 75.2 ± 2.8%, and all CT images could be acquired at the mid-diastolic phase.

**Table 1:** Characteristics of the patient population

**All patients (*n*** **= 83)**	
Age (years)	66.3 ± 8.6
Men, *n* (%)	35 (42%)
Height (cm)	170.6 ± 8.8
Body weight (kg)	80.2 ± 14.9
Body surface area (m^2^)	1.9 ± 0.2
Body mass index (kg/ m^2^)	27.6 ± 5.0
Hypertension, *n* (%)	10 (43%)
Dyslipidemia, *n* (%)	12 (52%)
Diabetes, *n* (%)	5 (22%)
Current smoking, *n* (%)	3 (13%)
**CT images acquisition conditions**	
Heart rate (beats/min)	59.8 ± 6.3
Time phase in the cardiac cycle during CT imaging	75.2 ± 2.8
Systolic blood pressure (mmHg)	146.3 ± 27.8
Diastolic blood pressure (mmHg)	84.4 ± 13.6
Atrial fibrillation, *n* (%)	8 (10%)
Interval between FFR_CT_ and invasive coronary angiography (days)	24.5 ± 19.5

### Distribution of distal FFR_CT_

The distribution of distal FFR_CT_ for each vessel is presented in Supplementary Figure 3. Compared to LCX, a lower proportion of FFR_CT_ > 0.80 was observed in LAD (54.2% vs. 89.2).

### Changes in FFR_CT_

**Fig. 2:**
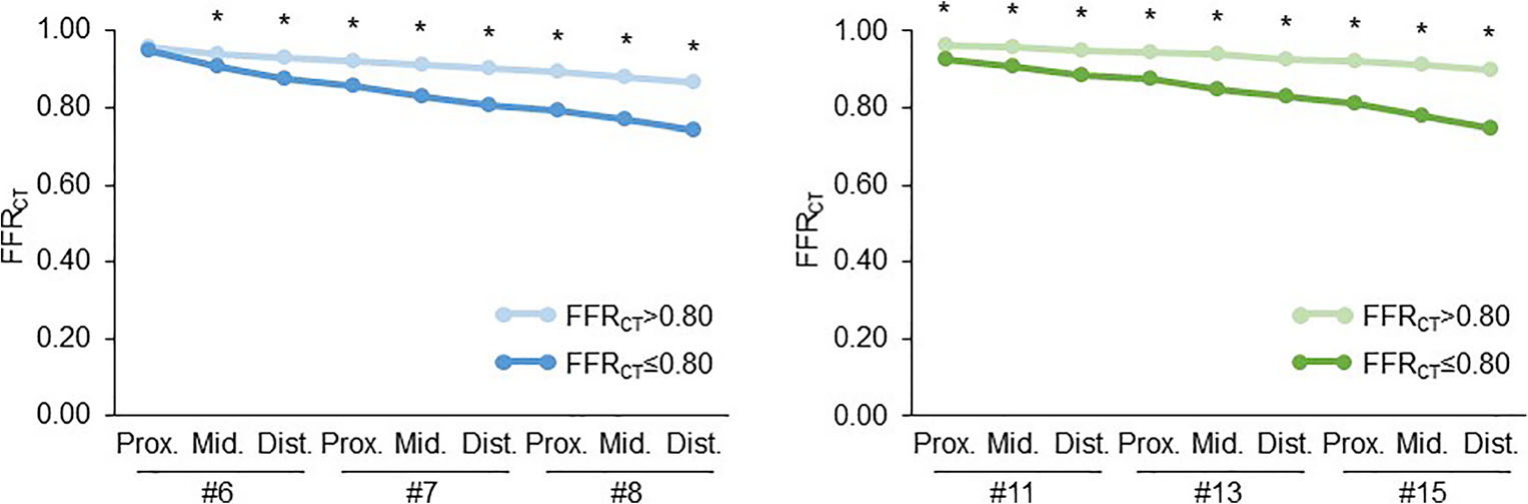
Changes in FFR_CT_ of LAD and LCX. Each coronary artery segment number is based on the American Heart Association classification. Dist., distal; Mid., middle; Prox., proximal. *p < 0.01 vs. FFR_CT_ ≤ 0.80

FFR_CT_ showed a continuous gradual decline from the proximal to the distal in LAD and LCX vessels in both FFR_CT_ > 0.80 and ≤ 0.80. LAD presented a more stepwise variation than LCX, and distal end of FFR_CT_ was significantly lower in LAD (0.81 ± 0.08 vs. 0.88 ± 0.06, *p* < 0.01). In LAD, proximal FFR_CT_ did not differ between FFR_CT_ > 0.80 and ≤ 0.80 (0.96 ± 0.02 vs. 0.95 ± 0.04, *p* > 0.05), but FFR_CT_ ≤ 0.80 showed a significant decline from the level of the middle segment 6 (0.94 ± 0.03 vs. 0.91 ± 0.07, *p* < 0.01), resulting in the distal end of FFR_CT_ to be significantly lower in FFR_CT_ ≤ 0.80 (0.86 ± 0.04 vs. 0.74 ± 0.07, *p* < 0.01). In LCX, FFR_CT_ ≤ 0.80 was significantly decreased at the proximal and distal end of FFR_CT_. ΔFFR_CT_ was significantly higher in LAD (0.14 ± 0.07) than in LCX (0.09 ± 0.05) (Table 2 and Figure 2).

**Table 2:** FFR_CT_ changes in LAD and LCX

	LAD (#6, #7, #8)			LCX (#11, #13, #15)		
	Total (*n* = 83)	FFR_CT_ > 0.80 (*n* = 45)	FFR_CT_ ≤ 0.80 (*n* = 38)	Total (*n* = 83)	FFR_CT_ > 0.80 (*n* = 74)	FFR_CT_ ≤ 0.80 (*n* = 9)
Proximal of #6 or #11	0.95 ± 0.03	0.96 ± 0.02	0.95 ± 0.04	0.96 ± 0.03	0.96 ± 0.02	0.93 ± 0.06^*^
Middle of #6 or #11	0.93 ± 0.05	0.94 ± 0.03	0.91 ± 0.07^*^	0.95 ± 0.03^†^	0.96 ± 0.02	0.91 ± 0.07^*^
Distal of #6 or #11	0.90 ± 0.06	0.93 ± 0.02	0.87 ± 0.08^*^	0.94 ± 0.04^†^	0.95 ± 0.03	0.89 ± 0.08^*^
Proximal of #7 or #13	0.89 ± 0.06	0.92 ± 0.02	0.86 ± 0.08^*^	0.94 ± 0.04^†^	0.95 ± 0.03	0.88 ± 0.07^*^
Middle of #7 or #13	0.87 ± 0.07	0.91 ± 0.03	0.83 ± 0.08^*^	0.93 ± 0.04^†^	0.94 ± 0.03	0.85 ± 0.07^*^
Distal of #7 or #13	0.86 ± 0.07	0.90 ± 0.03	0.81 ± 0.08^*^	0.92 ± 0.05^†^	0.93 ± 0.04	0.83 ± 0.07^*^
Proximal of #8 or #15	0.85 ± 0.07	0.89 ± 0.03	0.79 ± 0.08^*^	0.91 ± 0.05^†^	0.92 ± 0.04	0.81 ± 0.07^*^
Middle of #8 or #15	0.83 ± 0.08	0.88 ± 0.03	0.77 ± 0.07^*^	0.90 ± 0.06^†^	0.91 ± 0.05	0.78 ± 0.06^*^
Distal of #8 or #15	0.81 ± 0.08	0.86 ± 0.04	0.74 ± 0.07^*^	0.88 ± 0.06^†^	0.90 ± 0.04	0.75 ± 0.05^*^
ΔFFR_CT_	0.14 ± 0.07	0.09 ± 0.04	0.20 ± 0.06^*^	0.09 ± 0.05^†^	0.07 ± 0.04	0.18 ± 0.05^*^

*LAD* left anterior descending artery, *LCX* left circumflex artery. ^*^p < 0.01 vs. FFR_CT_ > 0.80. ^†^*p* < 0.01 vs. LAD

### Changes in FFR_CT_ according to vascular characteristics

Vessel length, lumen volume, and plaque characteristics including, low-attenuation plaque, intermediate-attenuation plaque, and calcified plaque volume were significantly higher in LAD. The bifurcation angle was significantly lower in the LAD angle (31.1 ± 6.6° vs. 42.8 ± 14.9°, *p* < 0.01). Both in LAD and LCX, bifurcation angle and vessel length were significantly higher in FFR_CT_ ≤ 0.80 (Table 3).

**Table 3:** Vessel morphology and composition of each vessel in LAD and LCX

	LAD			LCX		
	Total (*n* = 83)	FFR_CT_ > 0.80 (*n* = 45)	FFR_CT_ ≤ 0.80 (*n* = 38)	Total (*n* = 83)	FFR_CT_ > 0.80 (*n* = 74)	FFR_CT_ ≤ 0.80 (*n* = 9)
Bifurcation angle (°)	31.1 ± 6.6	29.4 ± 6.5	33.4 ± 6.0^*^	42.8 ± 14.9^†^	40.7 ± 13.7	59.5 ± 14.3^*^
Vessel length (mm)	106.8 ± 23.7	99.8 ± 24.4	115.4 ± 19.6^*^	80.1 ± 30.4^†^	77.5 ± 29.6	105.5 ± 23.3^*^
Lumen volume (mm^3^)	673.9 ± 231.3	661.1 ± 240.6	683.3 ± 218.4	547.9 ± 336.7^†^	527.9 ± 336.1	708.6 ± 268.5
LAP volume (mm^3^)	18.3 ± 20.5	14.8 ± 15.7	22.4 ± 24.5	8.1 ± 9.4^†^	7.4 ± 9.1	13.9 ± 10.1
IAP volume (mm^3^)	108.8 ± 86.6	98.1 ± 80.1	121.6 ± 93.2	48.1 ± 42.6^†^	43.1 ± 40.5	89.4 ± 38.4^*^
CP volume (mm^3^)	20.8 ± 28.4	18.1 ± 25.9	24.3 ± 30.5	2.5 ± 5.4^†^	1.8 ± 4.0	11.9 ± 15.4^*^

*CP* calcified plaque, *IAP* intermediate-attenuation plaque, *LAD* left anterior descending artery, *LAP* low-attenuation plaque, *LCX* left circumflex artery. ^*^p < 0.01 vs. FFR_CT_ > 0.80. ^†^*p* < 0.01 vs. FFR_CT_ > LAD

### Univariate and multivariate analysis of the relationship between FFR_CT_ and vessel characteristics

**Fig. 3:**
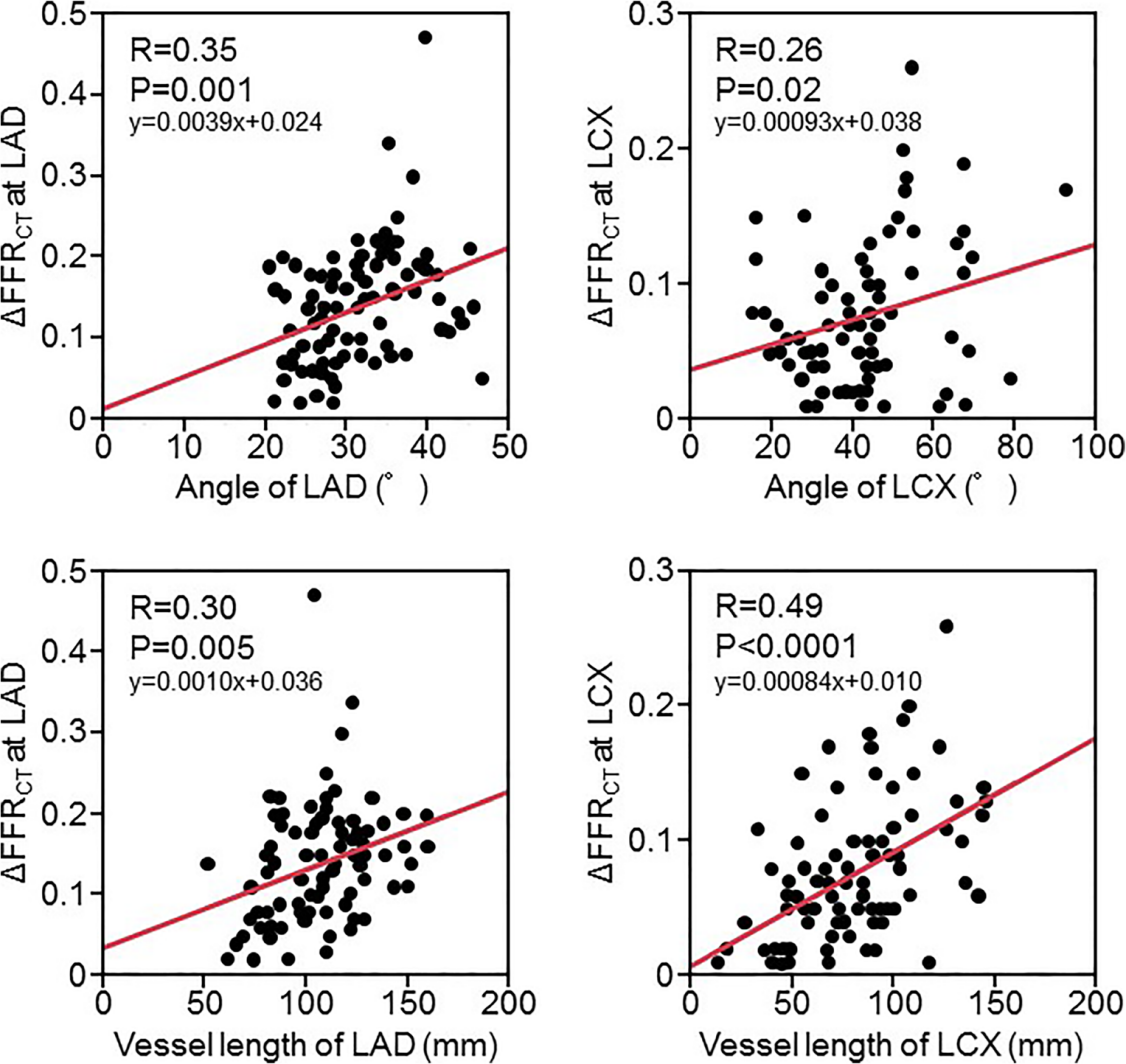
Relationship between ΔFFR_CT_, bifurcation angle, and vessel length. LAD, left anterior descending artery; LCX, left circumflex artery

In LAD, ΔFFR_CT_ significantly correlated with the bifurcation angle (*r* = 0.35, *p* = 0.001) and vessel length (*r* = 0.30, *p* = 0.005). With each 10° increase in bifurcation angle, FFR_CT_ changed by 0.063. In LCX, ΔFFR_CT_ also significantly correlated with the bifurcation angle (*r* = 0.26, *p =* 0.02), vessel length (*r* = 0.49, *p* < 0.0001). With each 10° increase in bifurcation angle, FFR_CT_ changed by 0.047 (Figure 3).

**Fig. 4:**
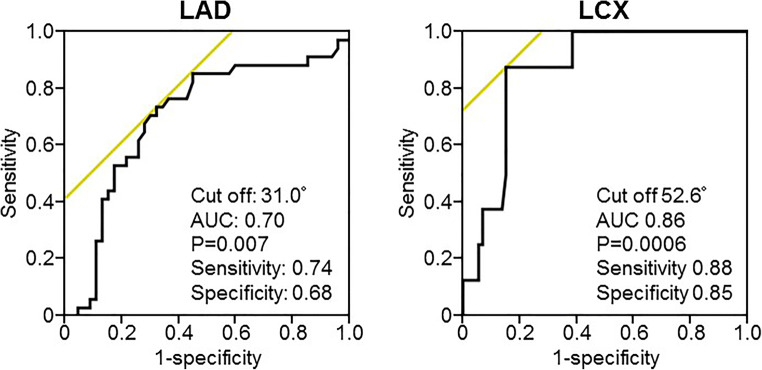
Receiver operating curve of bifurcation angle for predicting the distal vessel FFR_CT_ of ≤ 0.80. LAD, left anterior descending artery; LCX, left circumflex artery

Both angles of LAD- and LCX-related parameters were classified into three groups. The angles of LAD parameters were classified into the following groups: (vessel length and IAP volume) (LAP volume, ΔFFR_CT_, LAD angle, and CP volume) (lumen volume). The angles of LCX parameters were classified into the following groups: (vessel length, IAP volume, and LCX angle) (LAP volume, CP volume, and ΔFFR_CT_) (lumen volume) (Supplementary Figure 4). For LAD, multivariable analysis showed that angle (β-coefficient = 0.24, *p* = 0.02) and vessel length (β-coefficient = 0.55, *p* = 0.0003) had a predictive value for ΔFFR_CT_. For LCX, vessel length (β-coefficient = 0.46, *p* = 0.02) and calcified plaque volume (β-coefficient = 0.34, *p* = 0.001) were predictive for ΔFFR_CT_ (Table 4). Receiver operating characteristic curve revealed that both LAD and LCX angle had a predictive value for FFR_CT_ ≤ 0.80 at the distal aspect of the vessel (LAD; cut-off 31.0°, AUC 0.70, 95% CI 0.58–0.83, sensitivity 74%, specificity 68%, *p* = 0.007; LCX, cut-off 52.6°, AUC 0.86, 95% CI 0.76–0.96, sensitivity 88%, specificity 85%, *p* = 0.0006) (Figure 4).

**Table 4:** Univariable and multivariable analysis for ΔFFR_CT_

	Univariable analysis				Multivariable analysis			
	β	95%CI	t-value	*p* value	β	95%CI	t-value	*p* value
LAD								
LAD angle	0.35	0.2 to 0.6	3.35	0.001	0.24	0.04 to 0.5	2.37	0.02
Vessel length	0.30	0.03 to 0.2	2.81	0.006	0.55	0.08 to 0.3	3.81	0.0003
Lumen volume	−0.04	−0.009 to 0.006	-0.40	0.69				
LAP volume	0.10	−0.04 to 0.1	0.91	0.37				
IAP volume	0.14	−0.006 to 0.03	1.31	0.19				
CP volume	0.10	−0.03 to 0.09	0.89	0.37				
LCX								
LCX angle	0.26	0.01 to 0.2	2.47	0.02				
Vessel length	0.49	0.05 to 0.1	5.03	< 0.0001	0.46	0.01 to 0.1	2.41	0.02
Lumen volume	0.33	0.02 to 0.08	3.18	0.002				
LAP volume	0.36	0.08 to 0.3	3.48	0.0008				
IAP volume	0.44	0.02 to 0.07	4.36	< 0.0001				
CP volume	0.46	0.2 to 0.5	4.71	< 0.0001	0.34	0.1 to 0.4	3.33	0.001

*CI* confidence interval, *CP* calcified plaque, *IAP* intermediate-attenuation plaque, *LAD* left anterior descending artery, *LAP* low-attenuation plaque, *LCX* left circumflex artery

## Discussion

In patients with an ICA and CTA showing essentially vessels with no apparent CAD, our study highlighted the following: (1) Both LAD and LCX angles correlated with ΔFFR_CT_; (2) Bifurcation angle was one of the predictors of a distal FFR_CT_ ≤ 0.80 and an optimal cut-off value of 31.0° for the LAD and 52.6° for the LCX were demonstrated in our investigation.

To the best of our knowledge, few investigations have studied the role of the bifurcation angle on FFR_CT_ despite the fact that the bifurcation angle may even be one of the most important factors contributing to an unexpected FFR_CT_ decline in vessels with no apparent CAD. This has significant clinical implications.

There was some accordance between our study of bifurcation angles and previous articles. In previous studies that enrolled patients with suspected CAD, bifurcation angles of LAD and LCX assessed by CTA were 34.2 ± 13.4° and 44.3 ± 13.4° [8] and 37 ± 13° and 59 ± 21° [22], and assessed by ICA were 34.1 ± 18.5° and 42.8 ± 20.1° [19]. These bifurcation angles were larger than in our study where they were 31.1 ± 6.6° and 42.8 ± 14.9°, respectively. This difference might be due to selection bias. LAD angle was greater in patients with advanced calcification (33.8 ± 11.6°) than in those without (40.3 ± 10.0°) [14] LCX angle was also higher in patients with coronary stenosis ≥ 50% (39.5 ± 27.0°) than < 50% (46.9 ± 24.6°) [8].

An advantage of our investigation was that our results of the LAD and LCX angles were studied in patients with no apparent CAD. This is valuable as it shows that even with no apparent CAD the bifurcation angle has an influence on FFR_CT_. In the previous studies, many confounding factors could potentially have influenced the results.

The finding in our work that vessel morphology was the most important factor influencing FFR_CT_ decline was in line with previous studies [3]. However, we showed that the bifurcation angle also had a considerable influence on FFR_CT_ decline in vessels with no apparent CAD. We believe our findings are of clinical interest. As shown by different studies the important FFR_CT_ value should be placed in perspective and many factors should be taken into account when interpreting it. Both in LAD and LCX, vessel length was the strongest factor influencing FFR_CT_, followed by bifurcation angle in LAD, and CP volume in LCX. Experimental studies indeed showed that a wide bifurcation angle was closely associated with modifications of the bloodstream in vivo [23] as well as in vitro [22–24]. Fluid dynamically, a disturbance of laminar flow generates thermal energy, which is consumed, resulting in FFR_CT_ decline in not only atherosclerotic vessels but also normal vessels [10]. Turbulence may be an important factor in this setting. Previous investigations in patients with CAD with a wider bifurcation angle assessed by CTA [25], intravascular ultrasound [26], or angiography [19] showed a higher prevalence of CAD, high-risk plaque, or restenosis after stent implantation.

Our study is original in that it investigates the bifurcation angle in patients without CAD. The present study included only patients with no apparent CAD. In contradistinction, other studies have investigated the relationship between bifurcation angle and plaque formation. In these reports, bifurcation angle was shown to affect the shear stress and consequently influence plaque formation [27]. A wider bifurcation angle was shown to be related to higher turbulence and low shear stress which might cause plaque formation in the areas of bifurcation [24, 28]. Our study showed that plaque volume did not affect FFR_CT_ dynamics. We suggest that there was only limited total plaque volume to affect FFR_CT_ because of the enrolled patients with no apparent CAD. However, our results offer important additional information even in vessels with no apparent CAD the bifurcation angle has a significant effect on FFR_CT_ and erroneous clinical decisions could occur. Whether at a later phase of the disease, the bifurcation angle caused CAD was beyond the scope of our study.

In our study, the use of a cut-off value of 31.0° for LAD and 52.6° for LCX bifurcation after corrections for confounding factors, was still significant, suggesting that LAD and LCX angles were a reliable predictor of a distal vessel FFR_CT_ of ≤ 0.80.

To the best of our knowledge, this is the first work to investigate the effects of the left coronary bifurcation angle on FFR_CT_ in vessels with no apparent CAD. The present study included vessels with no apparent CAD to exclude confounding factors such as coronary stenosis. In common coronary artery disease, the effect of the bifurcation angle may be more important than the results of our study suggest. Our findings indicate that a large bifurcation angle can affect hemodynamics, resulting in the overestimation of FFR_CT_. FFR_CT_ should be interpreted more carefully and a correction may be required depending on the left coronary artery bifurcation angle for assessment of myocardial ischemia. Since the bifurcation angle has an effect, the addition of an angulation measurement to conventional FFR_CT_ evaluation may have clinical value and should be considered.

## Limitations

Our study has several limitations. First, this is a single-center study. Still, the number of patients included was sufficient for statistical analysis. Second, according to the American Heart Association [29] and European Society of Cardiology guidelines [30], FFR is recommended to assess angiographically intermediate-grade coronary stenosis. The present study included patients with vessels with < 20% coronary stenosis. Therefore, invasive procedures (measurement of FFR or bifurcation angle) were not performed. Previous studies have shown that FFR_CT_ can be an alternative test to invasive FFR due to the high concordance between FFR_CT_ and invasive FFR [31]. FFR values [31] and bifurcation angle [9, 32] assessed by CTA correlate well with invasive measurements. However, it is indeed uncertain whether distal FFR changes related to the bifurcation angle occur in invasive FFR as well as FFR_CT_. Third, despite the fact that only vessels with no apparent CAD were selected, plaque burden was still observed. Plaque burden observed with no apparent CAD may be due to the following factors: (1) Plaque formation generates at the bifurcation angle due to higher turbulence and low shear stress. The vessel diameter immediately after the bifurcation angle is large, resulting in higher absolute plaque volume even with no apparent CAD; (2) Diffuse plaque deposits throughout the vessel. (3) Plaque deposits on the extravascular side. Our plaque analysis software could not differentiate between intravascular and extravascular plaque. However, it is controversial and difficult to investigate FFR_CT_ in vessel models that completely eliminate the effects of plaque characteristics. Simulated vessel models are assumed to have a rigid wall rather than the elastic wall; therefore, such simulation does not reflect the physiological situation [23]. Plaque burden caused an impaired vasodilator capacity due to oxidative stress and inflammation [33, 34]. This vasodilator dysfunction could interfere with accurate assessment of FFR_CT_. Fourth, this study did not include ‘no obvious CAD’, thus minimal atherosclerotic lesions were present. Because vessels with advanced atherosclerosis also may show a complex morphology due to curvature or tortuosity in the actual clinical setting, measurement of the bifurcation would optimally be better performed by three-dimensional analysis. However, similar to our experience in previous studies [9, 18, 19, 25], it was quite difficult to assess the bifurcation angle by three-dimensional analysis. Moreover, complex vessel morphology may affect blood flow leading to paradoxical FFR_CT_ changes. Blood flow velocity is accelerated at the stenotic lesion, resulting in potential energy being converted into kinetic energy. A small amount of turbulent eddies generated at the stenotic lesion leads to less thermal energy loss, thus kinetic energy is reconverted into potential energy (pressure recovery phenomenon), which can cause FFR_CT_ increase In order to explore the influence of vessel morphology on FFR_CT_ changes, further investigations of various types of lesions in a larger number of patients may be needed. Fifth, the relationship between bifurcation angle, turbulence, and FFR_CT_ is not clear because turbulence is present not quantifiable [35].

## Conclusions

FFR_CT_ decline was dependent on each left coronary bifurcation angle. Vessel length most influences FFR_CT_, but also the bifurcation angle in vessels with no apparent CAD plays a significant role. As such, a correction may be required depending on the left coronary artery bifurcation angle when interpreting numerical values of FFR_CT_ for assessment of myocardial ischemia. We provide cut-off values at which this effect occurs.

## Supplementary information

ESM 1 (DOCX 780 kb)

## Abbreviations

CAD: Coronary artery disease
CTA: Computed tomography angiography
FFR: Fractional flow reserve
FFR_CT_: Computed tomography derived fractional flow reserve
LAD: Left anterior descending artery
LCX: Left circumflex artery

## Declarations

### Guarantor

The scientific guarantor of this publication is Johan De Mey.

### Statistics and biometry

No complex statistical methods were necessary for this paper.

### Methodology

• retrospective

• observational

• performed at one institution
