# Supplementary material for: Impact of coronary bifurcation angle on computed tomography derived fractional flow reserve in coronary vessels with no apparent coronary artery disease
Source: Eur Radiol. 2022 Sep 17;33(2):1277–85. doi: 10.1007/s00330-022-09125-3 (PMC9889442; doi:10.1007/s00330-022-09125-3)

**Supplementary** **Figure 1**

(A) FFR_CT_ measurement position. Coronary arteries are classified based on the American Heart Association classification and each segment is divided into three equal segments, proximal, middle, and distal, respectively. (B) Lumen volume measurement. Align the vessel to the region of interest on FFR_CT_ and vessel length and vessel composition (lumen volume and plaque volume) are measured semi-automatically.


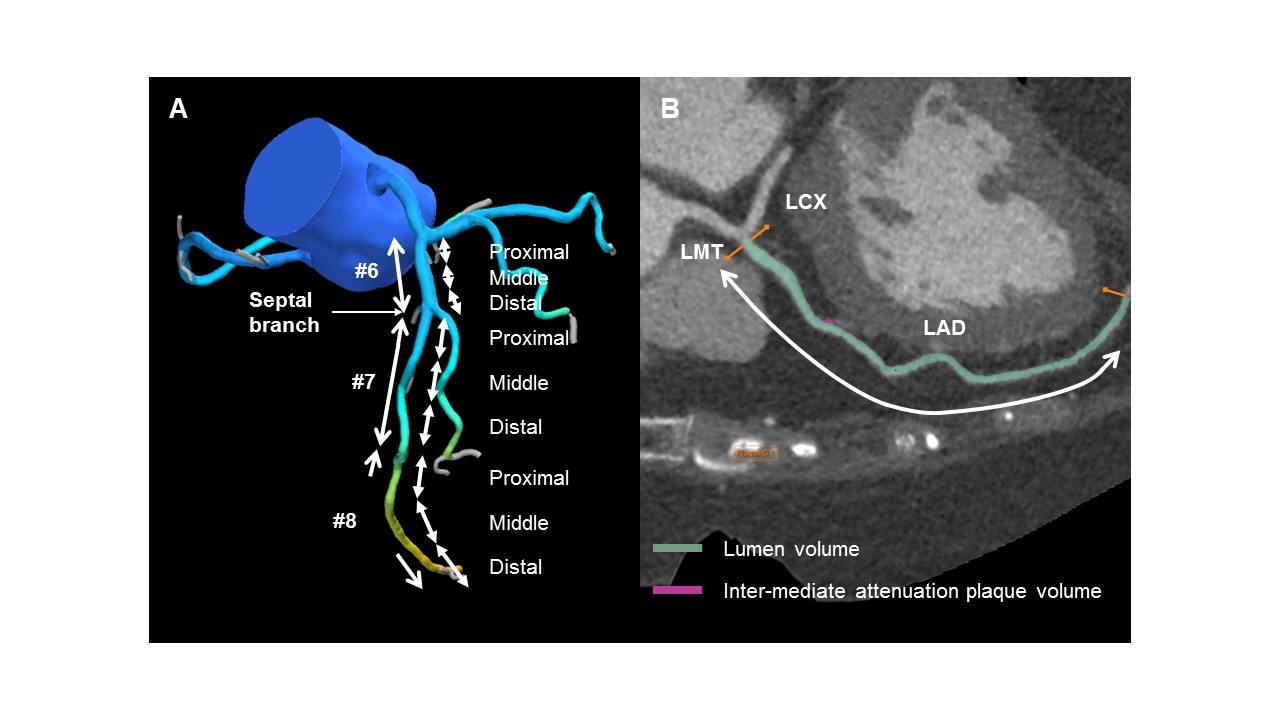


**Supplementary** **Figure 2**

Representative case of a wide bifurcation angle (upper panels) and a narrow bifurcation angle (lower panels). Three-dimensional volume rendered images (left panels). Bifurcation angle measurement (centre panels). FFR_CT_ (right panels). LAD, left anterior descending artery; LCX, left circumflex artery; LMT, left main trunk.


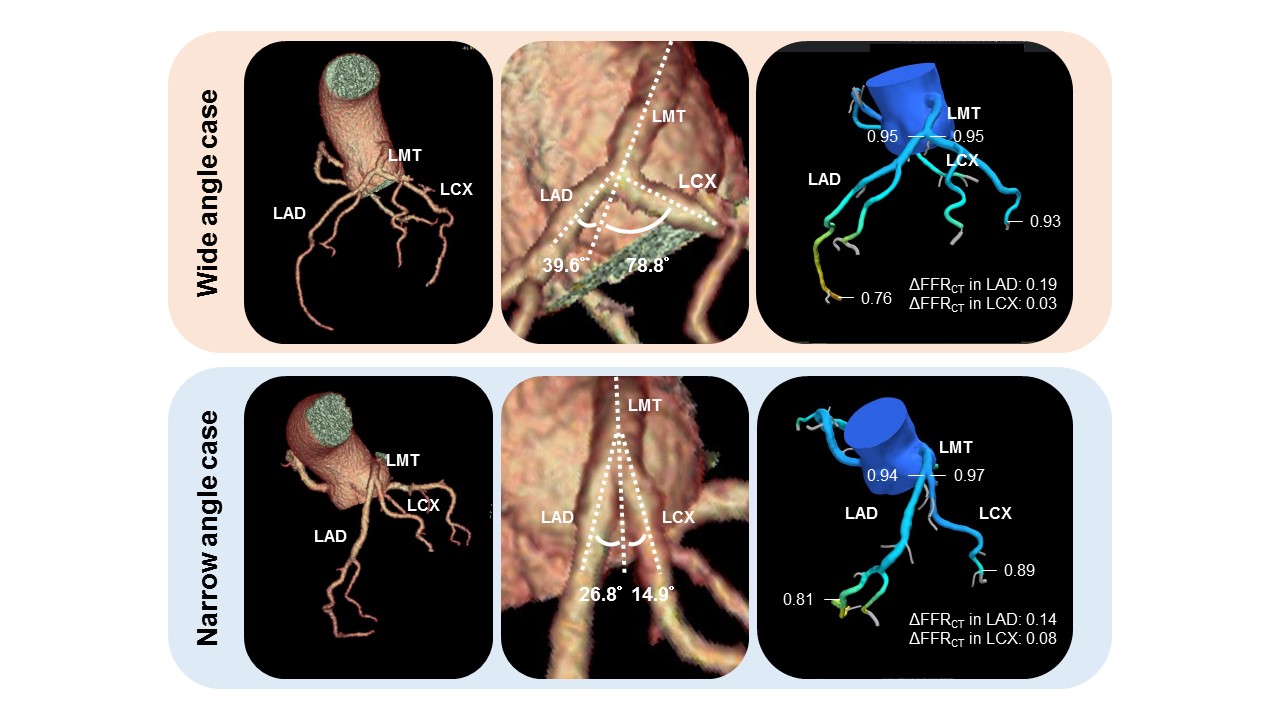


**Supplementary** **Figure 3**

Distribution of FFR_CT_ at distal LAD and LCX. LAD, left anterior descending artery; LCX, left circumflex.


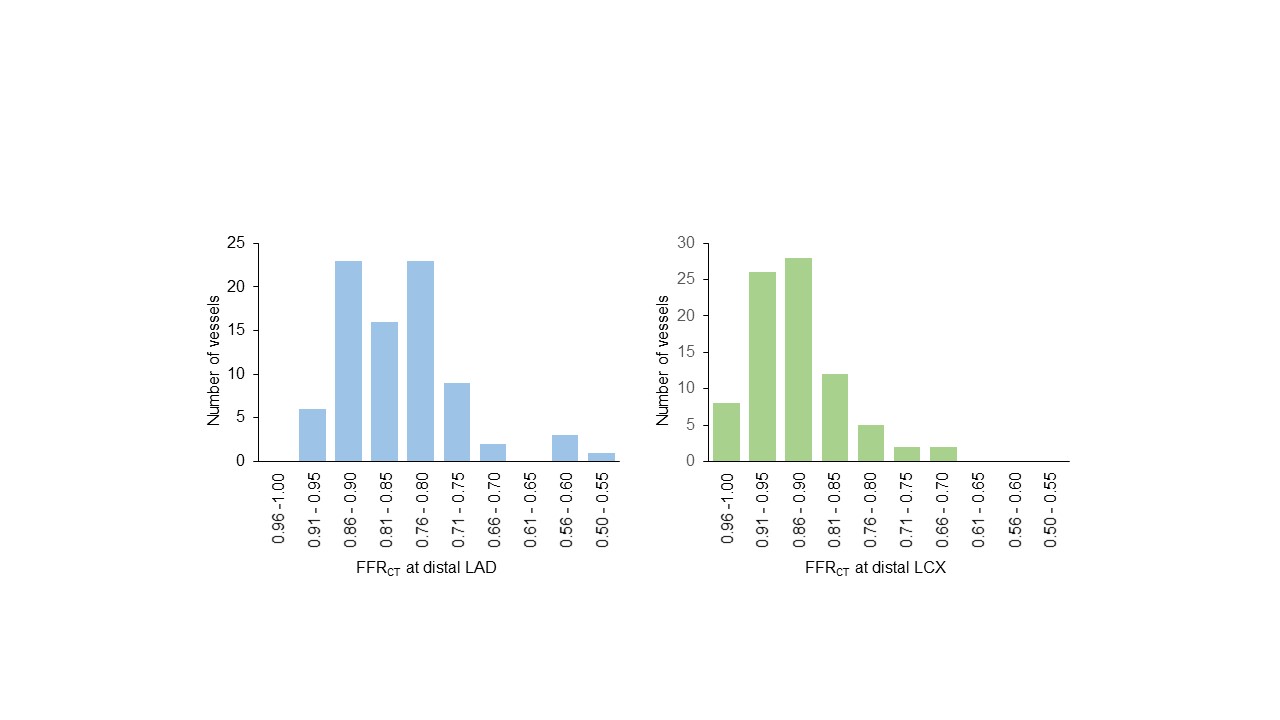


**Supplementary** **Figure 4**

Cluster dendogram of each bifurcation angle related parameters. (A) Angle of LAD. (B) Angle of LCX. CP, calcified plaque; IAP, intermediate-attenuation plaque; LAD, left anterior descending artery; LAP, low-attenuation plaque; LCX, left circumflex artery.


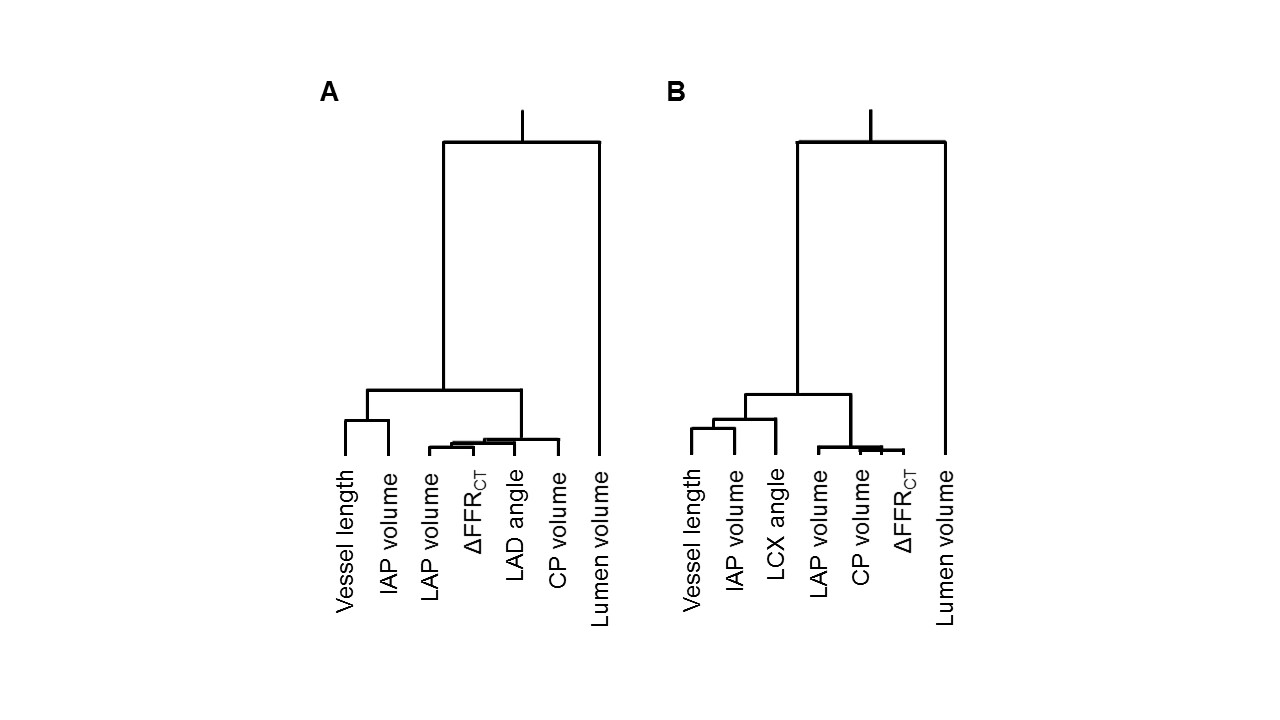

Supplement: Supplementary file 1 — (DOCX 780 kb) [file 330_2022_9125_MOESM1_ESM.docx]
